# Suppression of Problematic Compound Oligomerization by Cosolubilization of Nondetergent Sulfobetaines

**DOI:** 10.1002/cmdc.201500057

**Published:** 2015-03-11

**Authors:** Yumiko Mizukoshi, Koh Takeuchi, Misa Arutaki, Takeshi Takizawa, Hiroyuki Hanzawa, Hideo Takahashi, Ichio Shimada

**Affiliations:** [a] Graduate School of Pharmaceutical Science, The University of Tokyo7-3-1 Hongo, Bunkyo-ku, Tokyo 113-0033 (Japan); [b] Biomedicinal Information Research Center (BIRC) and Molecular Profiling Research Center (Molprof), National Institute of Advanced Industrial Science and Technology (AIST), 2-3-26 AomiKoto-ku, Tokyo 135-0064 (Japan); [c] Japan Biological Informatics Consortium (JBIC), 2-3-26 AomiKoto-ku, Tokyo 135-0064 (Japan); [d] Structural Biology Group Biological Research Department, Drug Discovery & Biomedical Technology Unit, Daiichi Sankyo RD Novare, 1-16-13 KitakasaiEdogawa-ku, Tokyo 134-8630 (Japan); [e] Graduate School of Medical Life Science, Yokohama City University, 1-7-29 Suehiro-chosurumi-ku, Yokohama 230-0045 (Japan)

**Keywords:** artifacts, drug discovery, NMR spectroscopy, nondetergent sulfobetaines, oligomerization

## Abstract

Numerous small organic compounds exist in equilibrium among monomers, soluble oligomers, and insoluble aggregates in aqueous solution. Compound aggregation is a major reason for false positives in drug screening, and even soluble oligomers can interfere with structural and biochemical analyses. However, an efficient way to manage the equilibrium of aggregation-prone compounds, especially those involved with soluble oligomers, has not been established. In this study, solution NMR spectroscopy was used as a suitable technique to detect compound oligomers in equilibrium, and it was demonstrated that cosolubilization of nondetergent sulfobetaines (NDSBs) can largely suppress compound oligomerization and aggregation by shifting the equilibrium toward the monomers. The rotational correlation time was obtained from the ratio of the selective and nonselective longitudinal NMR relaxation times, which directly and quantitatively reflected the apparent sizes of the compounds in the equilibrium. The rotational correlation time of the aggregation-prone compound SKF86002 (1 mm) was substantially reduced from 0.31 to 0.23 ns by cosolubilization of 100 mm NDSB195. NDSB cosolubilization allowed us to perform successful structural and biochemical experiments with substantially fewer artifacts, which represents a strategy to directly resolve the problematic oligomerization and aggregation of compounds.

## Introduction

**Figure 1:**
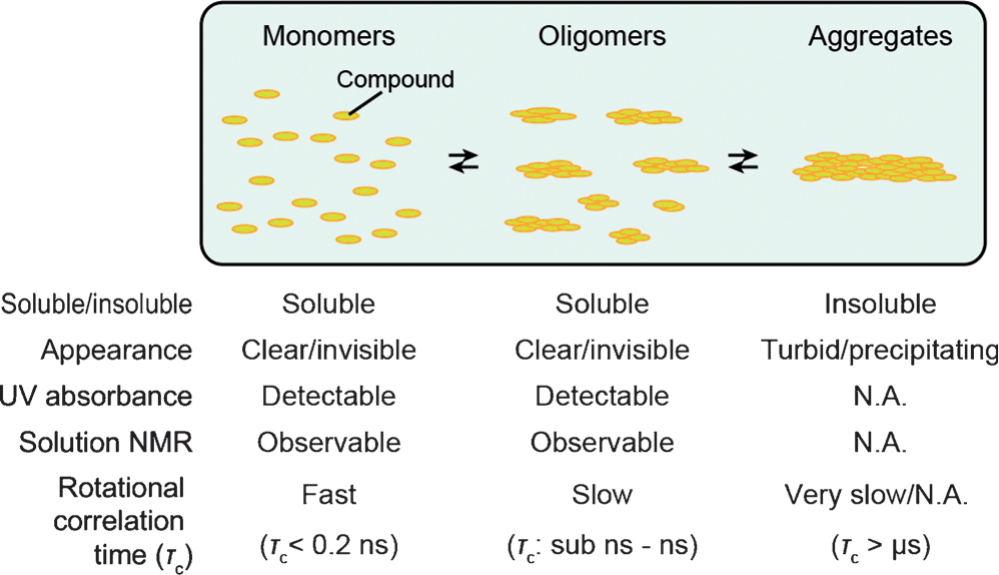
Three-phase equilibrium of the aggregation-prone compounds in aqueous solution. Schematic representation and properties of the monomers (left), soluble oligomers (middle), and insoluble aggregates (right) are shown. Note that these three states coexist in equilibrium in aqueous solution. N.A.: not applicable.

A number of small organic compounds, including those relevant for drug development, have the tendency to form aggregates in aqueous solution.[1–4] Such aggregation-prone compounds can adopt an equilibrium among three distinct states: monomers, soluble oligomers, and insoluble aggregates (Figure 1).[5,6]

Because of their small Stork radius, oligomers are soluble, invisible to the naked eye, and absorb UV light without extensive scattering. Therefore, distinguishing oligomers from monomers on the basis of visibility to the naked eye or by UV absorption is difficult. However, aggregates of compounds are rather obvious, because they are insoluble and appear turbid as a result of the scattering of visible light. Extensive aggregation often leads to spontaneous precipitation of the compounds. Otherwise, the aggregates can easily be sedimented by low-speed centrifugation (≈3000 g). After sedimentation, these aggregates no longer contribute to UV absorbance. The proportion of compound in each state within the above-mentioned equilibrium depends strongly on the experimental conditions. At low concentrations, the compounds are more in equilibrium between the two soluble states, that is, the monomers and oligomers, than between the soluble states and the insoluble aggregates. In contrast, higher concentrations favor aggregation. Often, the equilibrium is transient and is shifted toward aggregates in a time-dependent manner. This occurs on various timescales from seconds to days,[7] and in this case, the soluble compound oligomers are precursors to the aggregates. This behavior is analogous to that of fibril-forming proteins and peptides, such as α-synuclein and amyloid β, which form soluble oligomers prior to the formation of insoluble fibrils.[8]

Importantly, oligomers and/or aggregates may not exert the same activity as the monomers, because the active moiety within the compounds can be masked by molecular contacts within the oligomers and aggregates. Recent work has suggested that compound aggregates are a major reason for artificial inhibitions (false positives) in drug screening, because they segregate macromolecules from the experimental system.[3,9,10] Aggregation-prone compounds can be detrimental to high-throughput screening and chemical optimization of compounds, because they give erroneously high hit rates, poor structure–activity relationships, and/or insufficient specificity. Two major strategies have been developed to avoid these problems. One is to remove the aggregation-prone compounds from screening libraries, either in advance[11] or by following a screening procedure.[12] The other strategy is to perform an assay in the presence of a low-concentration detergent.[3] In the latter case, the presence of the low-concentration detergent masks the interaction surface between the aggregates and the macromolecules, which thereby prevents segregation of the macromolecules.[13] However, these strategies do not prevent aggregation itself.[3] In addition, the use of detergents at concentrations above their critical micelle concentration has been proposed to break up aggregates into soluble oligomers.[5,6,9] However, the high concentrations of detergents actually do not fully unravel the aggregates or oligomers into monomers.[5] Thus, in any case, the aggregation-prone compounds may not be selected for further development, even though their monomers may show genuine activity toward targets. Therefore, direct suppression of oligomerization and aggregation would be a more favorable resolution. In addition, monitoring the oligomerization and aggregation of compounds under the same conditions as those of the assays is of importance, because the equilibrium of aggregation-prone compounds can change extensively in the presence of proteins or other solutes. However, an efficient way to quantitatively analyze and control the equilibrium of these aggregation-prone compounds, especially those involved with soluble oligomers, has not been established thus far.

Solution NMR spectroscopy is a powerful technique in validating hit compounds and in developing high-affinity compounds in a structure-guided manner.[14–16] In solution NMR spectroscopy, both the monomers and oligomers of compounds are observable but the aggregates are not, because their huge apparent molecular weight and rotational correlation time (*τ*_c_>microseconds) make the relaxation of resonances from the aggregates too fast to be detected. More importantly, the populations of the two soluble states are reflected in the relaxation properties of the compounds, as the apparent *τ*_c_ of the compound oligomers is substantially larger (sub-nanoseconds to nanoseconds) than that of the monomers (apparent *τ*_c_<0.2 ns, Figure 1). Therefore, solution NMR spectroscopy can be used to define the equilibrium of the aggregation-prone compounds, especially those involving soluble oligomers.[5,6,12,17]

In this study, we propose that the ratio of selective and nonselective longitudinal relaxation times (*T*_1_^s^/*T*_1_^ns^),[18] which directly reflects the apparent *τ*_c_, is a sensitive metric for detecting the oligomer in the equilibrium with the aggregation-prone compounds. By using solution NMR spectroscopy and other biochemical techniques, we found that nondetergent sulfobetaines (NDSBs)[19,20] are potential cosolubilization agents that have the ability to dissolve the unwanted oligomers and aggregates by shifting the equilibrium toward the monomers. NDSB cosolubilization leads to successful NMR spectroscopy measurements and biochemical assays, and it can be used widely in the field of structure-guided drug development.

## Results and Discussion

**Figure 2:**
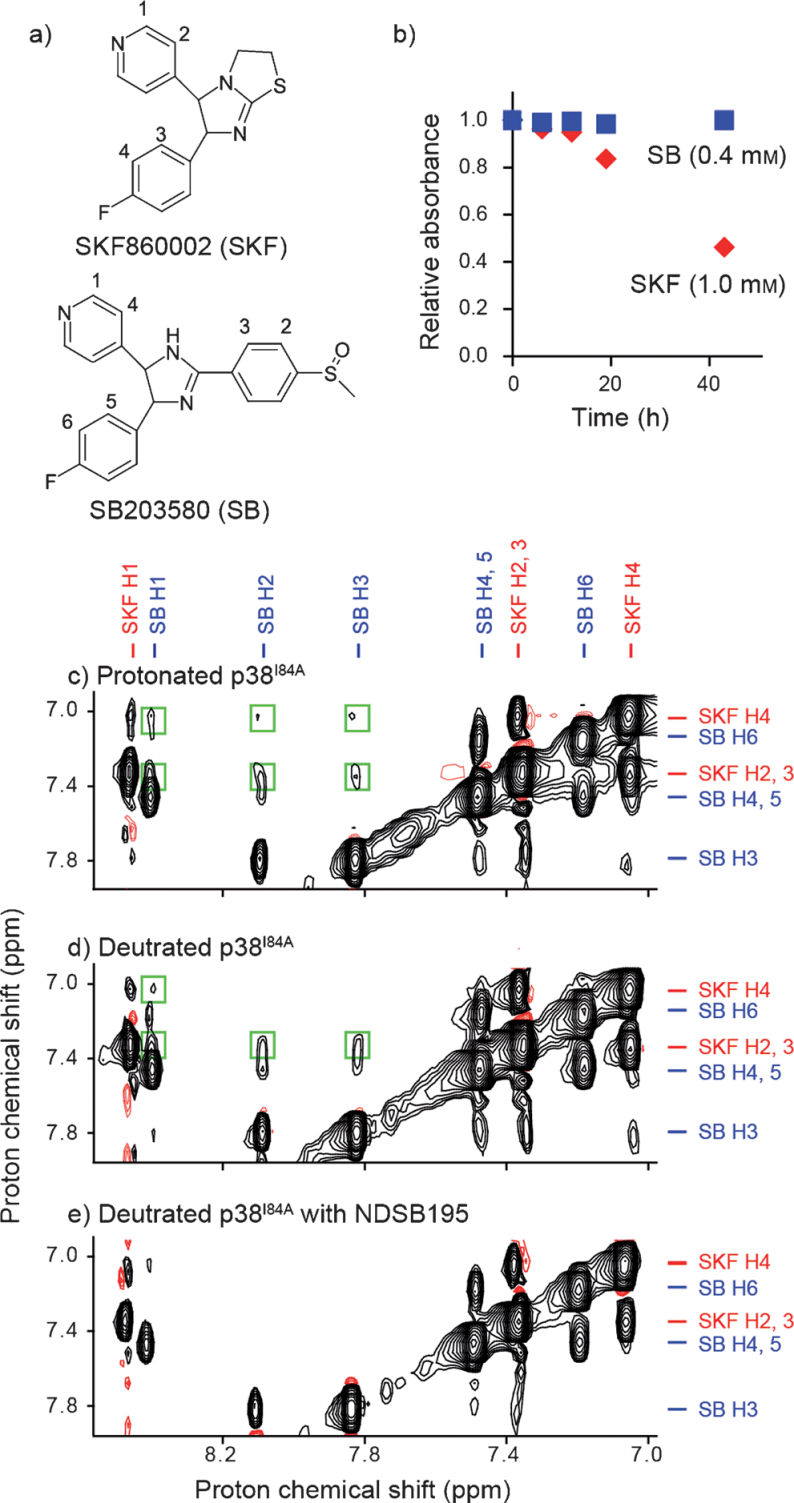
Biochemical and structural analyses of aggregation-prone SKF and soluble SB. a) Chemical structures of SKF and SB. b) Time-dependent change in the relative UV absorbance of 1.0 mm SKF and 0.4 mm SB solutions measured at *λ*=324 and 314 nm, respectively. c–e) INPHARMA NOESY spectra of SKF, SB, and p38α^I84A^ mixtures. p38α^I84A^ was protonated in c) and deuterated in d, e). In e), 100 mm NDSB195 was added to the sample. Mixing time was set to 200 ms. Interligand NOE cross-peaks are indicated by green boxes.

SKF86002 (SKF, Figure 2 a) is an inhibitor of human mitogen-activated protein kinase p38α; it shows inhibitory constant (*K*_i_) values of approximately 0.5 μm.[21] SKF shows characteristics of an aggregation-prone compound if dissolved at high concentrations. Figure 2 b shows the time-dependent change in absorbance at *λ*=324 nm of a 1.0 mm aqueous solution of SKF incubated at 25 °C. The soluble SKF fraction, which corresponds to the absorbance at *λ*=324 nm, decreases after 12 h. The soluble SKF fraction further decreases to approximately 40 % of its initial value at 43 h after sample preparation. This decrease in UV absorbance with a delay period is typical for compounds that show equilibrium between monomers and soluble oligomers before forming insoluble aggregates.

Upon mixing 1.0 mm SKF and the competing soluble ligand SB203580 (SB, 0.4 mm; Figure 2 a)[22] with 25 μm of the I84 A mutant of p38α (p38α^I84A^), which shows free-bound exchange properties suitable for an interligand nuclear Overhauser effect (NOE) for pharmacophore mapping (INPHARMA) experiments,[15,16,23] a substantial number of artificial NOE cross-peaks were observed in the spectrum (Figure 2 c). Under the experimental conditions, interligand NOE cross-peaks are observed for almost all proton pairs between the SKF and SB compounds. This is atypical of an INPHARMA experiment, which is expected to specify the shared binding epitopes by indirect interligand NOEs through protein protons.[15] These interligand NOEs are observed even for experiments in which deuterated p38α^I84A^ is used (Figure 2 d). This observation clearly indicates that these interligand NOEs are artificial and are not from indirect magnetization transfer between the shared binding epitopes. Interestingly, these interligand NOEs are not observed in a NOESY spectrum of the SKF and SB mixture without p38α^I84A^ (Figure S1, Supporting Information). Because the efficiency of interligand NOEs depends on the apparent *τ*_c_ of the observing molecule,[24] these data suggest that the oligomers are not large enough to give artificial NOEs in the absence of the protein. However, in the presence of the protein, transient attachment to the protein surfaces would make the oligomers have sufficient apparent *τ*_c_ to cause artificial NOEs. It should also be noted that no aggregation is detected in the INPHARMA experiments.

**Figure 3:**
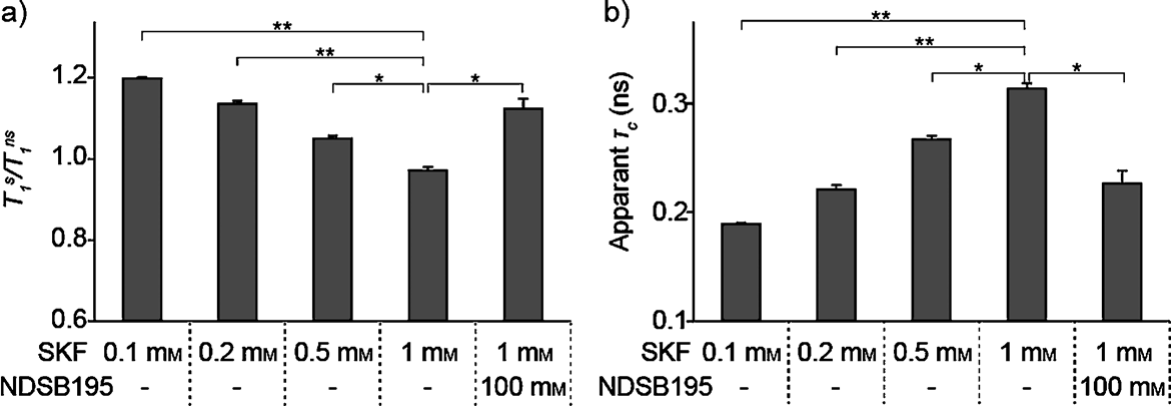
*T*_1_^s^/*T*_1_^ns^ and apparent *τ*_c_ values of SKF under various conditions. a) The *T*_1_^s^/*T*_1_^ns^ value of the H1 proton of SKF and b) the apparent *τ*_c_ under various conditions. *: *P*<0.001, **: *P*<0.0001.

To investigate if the soluble oligomers exist before aggregate formation, the *T*_1_^s^/*T*_1_^ns^ values and the apparent *τ*_c_ were calculated for various concentrations of SKF.[18] The *T*_1_^s^/*T*_1_^ns^ and apparent *τ*_c_ values of SKF at each concentration are shown in Figure 3 and Table 1. The *T*_1_^s^/*T*_1_^ns^ values decrease in a concentration-dependent manner, which indicates that the apparent *τ*_c_ of SKF in solution is larger at higher concentrations. In addition, soluble oligomers are already formed for concentrations at which the aggregates are not detected. Thus, the *T*_1_^s^/*T*_1_^ns^ experiment is suitable to monitor the presence of soluble oligomers in the equilibrium. Although, there is an NMR spectroscopy strategy to detect oligomers from concentration-dependent changes in linewidth or chemical shifts,[5,6] the use of the strategy is rather limited to aromatic compounds, which are expected to induce strong ring current shifts with each other. In addition, the amount of change in the chemical shift would strongly depend on the structural architecture of the oligomers. In addition, WaterLOGSY and selective T_1_ methods have been proposed as quality-control experiments to detect oligomers.[12] However, the WaterLOGSY method is not sensitive to oligomers with relatively small apparent molecular weights. The selective T_1_ experiment will resolve the molecular weight problem; however, the WaterLOGSY and selective T_1_ experiments are both rather qualitative in estimating the existence of the oligomerization and aggregation states. In contrast, the *T*_1_^s^/*T*_1_^ns^ method proposed herein is not limited by the chemical structures of the compounds, and the *τ*_c_ values deduced from the *T*_1_^s^/*T*_1_^ns^ method would directly and quantitatively reflect the apparent sizes of the compounds in aqueous solution. In addition, the *T*_1_^s^/*T*_1_^ns^ values are most sensitive to changes in *τ*_c_ values ranging from 10/*ω*>*τ*_c_>0.1/*ω* (in which *ω* is the proton Larmor frequency), which corresponds to an apparent *τ*_c_ of 0.16–16 ns for a 600 MHz spectrometer (Figure S2, Supporting Information).[18] Indeed, we also applied WaterLOGSY to our system; however, we failed to detect the WaterLOGSY signals originating from the SKF oligomer. Considering the fact that the NOE signals derived from SFK are not observed in the NOESY spectrum under the same conditions (Figure S1, Supporting Information), the apparent molecular weight of the SKF oligomer is within the range of the correlation times for which strong NOE and WaterLOGSY signals are not observed. In agreement with this notion, the *T*_1_^s^/*T*_1_^ns^ and *ωτ*_c_ values of SKF in solution are close to 1. Thus, in comparison with the previous strategies, the *T*_1_^s^/*T*_1_^ns^ method has wider applicability and quantitativity and is indispensable for characterizing detrimental soluble oligomers in solution. In particular, some inhibitors with lower *K_i_* values oligomerize at NMR spectroscopy observable concentrations but remain as monomers around their functionally effective concentrations. In this case, indirect NMR spectroscopy methods to detect low-concentration oligomers or aggregates by a cosolubilized high-concentration probe compound would be effective.[12] Notably, the *T*_1_^s^/*T*_1_^ns^ method can also be extended in the same way, although it will lose quantitativity.

**Table 1:** *T*_1_^s^, *T*_1_^ns^, *T*_1_^s^/*T*_1_^ns^, and apparent *τ*_c_ of the H1 proton of SKF under various conditions.[a]

SKF [mm]	NDSB195 [mm]	*T*_1_^s^ [s]	*T*_1_^ns^ [s]	*T*_1_^s^/*T*_1_^ns^	*τ_c_* [ns]
0.1	–	2.77±0.01	2.31±0.01	1.20±0.00	0.189±0.001
0.2	–	2.70±0.02	2.38±0.02	1.14±0.01	0.221±0.004
0.5	–	2.46±0.03	2.32±0.01	1.05±0.01	0.267±0.003
1.0	–	2.27±0.02	2.34±0.01	0.97±0.08	0.313±0.005
1.0	100	2.65±0.07	2.36±0.02	1.13±0.02	0.226±0.012

[a] Values are the mean ±standard error of at least three experiments.

**Figure 4:**
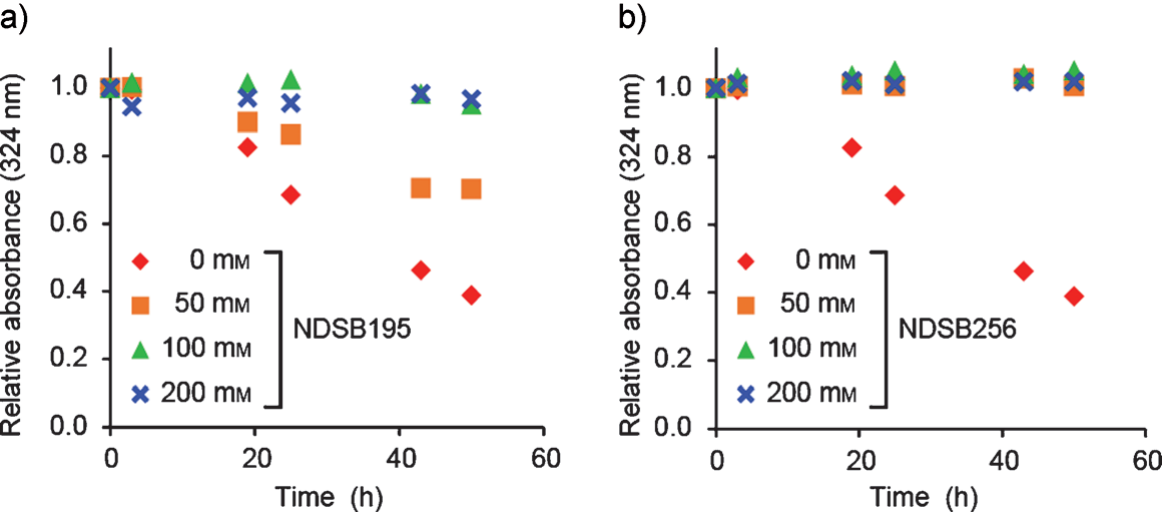
Time-dependent change in the UV absorbance of a SKF solution with and without NDSB cosolubilization. UV absorbance (*λ*=324 nm) of a 1.0 mm SKF solution at the indicated concentration of a) NDSB195 and b) NDSB256. Relative absorbance of each sample is plotted against time after sample preparation.

To suppress unwanted oligomerization and aggregation of compounds by shifting the equilibrium toward the monomers, we tried to find additives to inhibit SKF oligomerization and aggregation. We tested additives that are known to improve protein solubility: arginine[25] and NDSBs. For the NDSBs, choline-O-sulfate (COS),[26] NDSB195, and NDSB256[19,20] were tested. The changes in UV absorbance at *λ*=324 nm were monitored for up to 48 h after sample preparation to analyze the extent of aggregation. The aggregation of SKF was significantly inhibited by the addition of NDSB195 and NDSB256 (Figure 4) but was unaffected by arginine and COS (Figure S3, Supporting Information). In the presence of 100 mm NDSB195, the *T*_1_^s^/*T*_1_^ns^ value of SKF at 1 mm concentration was improved from 0.97 to 1.1, and the apparent *τ*_c_ was reduced from 0.31 to 0.23 ns (Figure 3 and Table 1). In addition, the artificial interligand NOEs in the INPHARMA NOESY spectrum with deuterated p38α^I84A^ (Figure 2 d) were substantially suppressed by the presence of 100 mm NDSB195 (Figure 2 e).

These data indicate that cosolubilization of NDSBs effectively relieves both oligomerization and aggregation by shifting the equilibrium toward the monomers. It has been shown that NDSBs prevent protein aggregation by competing for abortive electrostatic and hydrophobic interactions.[19,20] Because oligomerization and aggregation are largely driven by hydrophobic interactions,[4] it should be reasonable that NDSB256, which is the most hydrophobic among the tested NDSBs, would be the most effective in preventing the formation of oligomers and aggregates. Although NDSB256 at a high concentration might nonspecifically interact with the aggregation-prone compounds, such interactions can easily be detected through chemical shift perturbations of the compounds. In such a case, we would recommend the use of NDSB195, which might be less effective, but it has a lower chance of showing nonspecific interactions. It has been reported that protein resonances are not significantly perturbed at a NDSB concentration of 100 mm,[20] and these are the same conditions used in the present study. In addition, we confirmed that NDSBs did not perturb the resonances of the compound (Figure S4, Supporting Information), or those of p38α under our experimental conditions (data not shown). Thus, the relief of oligomerization and aggregation by cosolubilization of NDSBs does not rely on the specific interaction of the NDSBs with the compounds or proteins. It has been proposed that cosolubilization with nonionic detergents, such as Tween-80, can change the equilibrium of the aggregation-prone compounds and dissolve insoluble aggregates.[5,6,9] However, the addition of Tween-80 to a mixture of the SKF and SB compounds at the reported concentration (1.3 mm)[5] resulted in the emergence of strong negative NOEs in the NOESY spectra (Figure S4, Supporting Information), and a lower concentration of Tween-80 (0.01 mm), which was used in the previous publication,[3] induced negative NOEs. Thus, unlike NDSBs, the detergent cannot monomerize the aggregation-prone compounds. Therefore, NDSBs would be preferable reagents in structure–activity relationship studies, which require a higher level of uniformity in the compound solubilization states.

**Figure 5:**
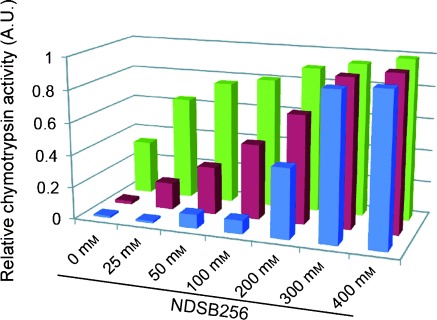
NDSB cosolubilization relieves the artificial inhibition of enzymatic activity. The aggregation-prone compounds, I4PTH (0.2 mm; ▪), clotrimazole (0.4 mm; ▪), and benzyl benzoate (1.0 mm; ▪), were added to the reaction solutions of the chymotrypsin assays with the indicated concentrations of NDSB256. The bars indicate the amount of product in the presence of the aggregation-prone compounds relative to that in the absence of the aggregation-prone compounds.

We found that cosolubilization of the NDSBs also prevented aggregation of other well-known aggregation-prone compounds. For tetraiodophenolphthalein (I4PTH),[4] the absorbance at *λ*=316 nm, which is specific to I4PTH, was increased in the presence of NDSB256 (Figure S5, Supporting Information). In addition, promiscuous inhibition of chymotrypsin activity by the aggregation-prone compounds benzyl benzoate, clotrimazole, and I4PTH was largely relieved by NDSB256 cosolubilization (Figure 5).[2,3] We performed a *T*_1_^s^/*T*_1_^ns^ experiment with 1.0 mm benzyl benzoate cosolubilized with 100 mm NDSB256. In the experiment, the *T*_1_^s^/*T*_1_^ns^ value of benzyl benzoate was 1.02. This value indicates that benzyl benzoate is not fully monomerized with 100 mm NDSB, owing to its stronger tendency for oligomerization and aggregation; this is consistent with the fact that the activity of chymotrypsin in the enzymatic assay was not fully recovered at this NDSB concentration (Figure 5). Notably, the *T*_1_^s^/*T*_1_^ns^ value for benzyl benzoate was 1.00 with NDSB195 cosolubilization. The consistent *T*_1_^s^/*T*_1_^ns^ values indicate that the *T*_1_^s^/*T*_1_^ns^ experiment is not perturbed by the strong aromatic resonances originating from NDSB256, except for the case in which there is signal degeneracy.

## Conclusions

In this study, we showed that the ratio of selective and nonselective longitudinal relaxation times and the apparent rotational correlation time deduced from solution NMR spectroscopy measurements were sensitive in detecting soluble oligomers of aggregation-prone compounds. In addition, we found that cosolubilization of nondetergent sulfobetaines (NDSBs) reduced oligomerization and subsequent aggregation of compounds by shifting the equilibrium toward the monomers. We successfully reduced the artifacts in solution NMR spectroscopy and biochemical assays by NDSB cosolubilization. Therefore, NDSBs represent a class of cosolubilization reagents that directly resolve the oligomerization and aggregation of compounds for successful structural and biological analyses.

## Experimental Section

### Materials

SK86002, SB203580, NDSB195, and NDSB256 were purchased from Calbiochem (Darmstadt, Germany). COS was purchased from Cambridge Isotope Laboratories. I4PTH and benzyl benzoate were purchased from TCI (Tokyo, Japan). Clotrimazole was purchased from Wako (Tokyo, Japan). Arginine hydrochloride, chymotrypsin, the photometric substrate *N*-succinyl-Ala-Ala-Pro-Phe *p*-nitroanilide, and Tween-80 were purchased from Sigma–Aldrich. According to the provider’s information, all chemicals were confirmed to have more than 95 % purity in HPLC or TLC.

### Protein production

The cDNA of human p38α (2–360) was cloned into the pET15b vector (Novagen, Madison, USA), and the I84A mutation was introduced by QuikChange mutagenesis (Agilent technologies) by using the vendor-provided protocol. p38α^I84A^ was expressed in *E. coli* cells, BL21(DE3) strain. The *E. coli* cells that harbor the p38α^I84A^ expression plasmids were grown in H_2_O or D_2_O M9 media supplemented with appropriate stable isotopes. The recombinant protein was purified by Ni-NTA resin affinity chromatography and size- exclusion chromatography, as published elsewhere.[27]

### NMR spectroscopy experiments

INPHARMA experiments were performed with 25 μm p38α^I84A^ in 25 mm deuterated Tris-buffer (pH 7.2) in D_2_O containing 150 mm NaCl and 1 mm deuterated dithiothreitol (DTT) at 25 °C with a Bruker Avance 600 MHz spectrometer equipped with a cryocooled triple resonance (TXI) probe. The p38α inhibitors (SKF and SB) were prepared in deuterated dimethyl sulfoxide (DMSO) and were added directly to the protein solution. The final concentrations of SKF and SB were 1.0 and 0.4 mm, respectively. The final concentration of deuterated DMSO was 10 % (*v*/*v*). NDSBs were prepared as 1 m stock solutions and were added to the protein solution prior to the addition of the ligands. Tween-80 was prepared as 10 % (*v*/*v*) and was diluted to a final concentration of 1.3 or 0.01 mm. 2048 and 256 points were recorded for the direct and indirect dimensions, respectively. The number of scans was set to 64, and the repetition delay was 1.2 s. Mixing time was set to 0.2 s. The typical experimental time was 7.5 h.

*T*_1_^s^ and *T*_1_^ns^ experiments for SKF were performed in 25 mm deuterated Tris-buffer (pH 7.2) in D_2_O containing 150 mm NaCl and 10 % (*v*/*v*) deuterated DMSO at 25 °C, and *T*_1_^s^ and *T*_1_^ns^ experiments for benzyl benzoate were performed in 50 mm potassium phosphate buffer (pH 7.0) in D_2_O containing 5 % (*v*/*v*) deuterated DMSO at 25 °C with a Bruker Avance 600 MHz spectrometer equipped with a cryocooled TXI probe. Selective inversion was performed with a Gaussian-selective pulse (12.5 Hz), whereas nonselective inversion was performed with a rectangular hard pulse (16.7 kHz). 8192 points were recorded for the direct dimension. The number of scans was set to 8, but 64 and 16 scans were used under the 0.1 mm SKF and 0.2 mm SKF conditions, respectively. The repetition delay was 15 s. The inversion recovery delays were set to 0.01, 0.25, 0.5, 0.8, 1.2, 1.6, 2.0, 3.0, 4.0, 5.0, and 6.0 s. The typical experimental time was 1 h. The *τ*_c_ values were calculated from selective (*T*^s^_1i_) and nonselective (*T*^ns^_1i_) proton spin-lattice relaxation times by assuming a pure dipolar mechanism according to Equations (1)–(4):[18]1, 2, 3, 4

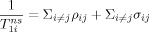
(1)

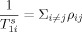
(2)

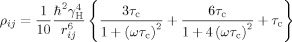
(3)

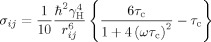
(4)

in which *ρ_ij_* and *σ_ij_* are the autorelaxation and cross-relaxation terms for a proton pair, respectively; *r*_*ij*,_ *ω*, *ħ*, and *γ* are the distance between *i*^th^ and *j*^th^ protons, the proton Larmor frequency, the reduced Planck constant, and the gyromagnetic ratio, respectively.

All NMR spectra were processed with the Topspin 2.1 program (Bruker) and were analyzed with Sparky (http://www.cgl.ucsf.edu/home/sparky/).

### UV absorbance spectroscopy

The UV absorbance of the SKF and SB compounds was measured at *λ*=324 and 314 nm, respectively, in 25 mm deuterated Tris-buffer (pH 7.2) in D_2_O containing 150 mm NaCl and 10 % (*v*/*v*) deuterated DMSO at 25 °C. The UV absorbance of I4PTH was measured at *λ*=316 nm with 50 mm potassium phosphate buffer (pH 7.0) in H_2_O at 25 °C. Prior to the UV measurements, the samples were centrifuged at 3000 *g* for 5 min to sediment the compound aggregates.

### Chymotrypsin assay

Assays were performed in 50 mm potassium phosphate buffer (pH 7.0) at 25 °C. Stocks of inhibitors were prepared in DMSO, and the final concentration of the inhibitors was 0.2, 0.4, and 1.0 mm for I4PTH, clotrimazole, and benzyl benzoate, respectively. No more than 5 % DMSO was present in the assay. The NDSB was directly added to the assay buffer. Chymotrypsin (28 nm) was incubated with the inhibitor for 5 min before the reaction, and the reaction was initiated by the addition of 200 μm photometric substrate, *N*-succinyl-Ala-Ala-Pro-Phe *p*-nitroanilide. The progress of the reaction was monitored by the absorbance at *λ*=405 nm.[2]

## Notes

Supporting Information for this article is available on the WWW under http://dx.doi.org/10.1002/cmdc.201500057. It contains the NOESY spectrum of the SKF/SB mixture, the dependence of the T1 s/T1 ns values on wtc, the time-dependent changes in the UV absorbance of the SKF solution with arginine or COS, the effect of NDSB or Tween-80 on the NOESY spectra of the SKF/SB mixture, and the relief of I4PTH aggregation by NDSB256 cosolubilization.
