# Supplementary material for: Suppression of Problematic Compound Oligomerization by Cosolubilization of Nondetergent Sulfobetaines
Source: ChemMedChem. 2015 Mar 11;10(4):736–41. doi: 10.1002/cmdc.201500057 (PMC4471626; doi:10.1002/cmdc.201500057)
Supplement: Supplementary file 1 [file cmdc0010-0736-sd1.pdf]

CHEMMEDCHEM
Chemistry & Drug Discovery

Supporting Information

Suppression of Problematic Compound Oligomerization by Cosolubilization of Nondetergent Sulfobetaines

Yumiko Mizukoshi,[b, c] Koh Takeuchi,[b] Misa Arutaki,[b, c] Takeshi Takizawa,[d]
Hiroyuki Hanzawa,[d] Hideo Takahashi,*[b, e] and Ichio Shimada*[a, b]

cmdc_201500057_sm_miscellaneous_information.pdf

Supporting Information

Contents

Figure S1: NOESY spectrum of SKF and SB mixture.

Figure S2: Dependence of the T_1^s/T_1^{ns} values on $\omega\cdot\tau_c$.

Figure S3: Time-dependent changes in UV absorbance of SKF solution with indicated amount of (a) arginine and (b) COS.

Figure S4: Effect of NDSB or Tween 80 on the NOESY spectra of the SKF SB mixture.

Figure S5: Relief of I4PTH aggregation by NDSB256 co-solubilization.

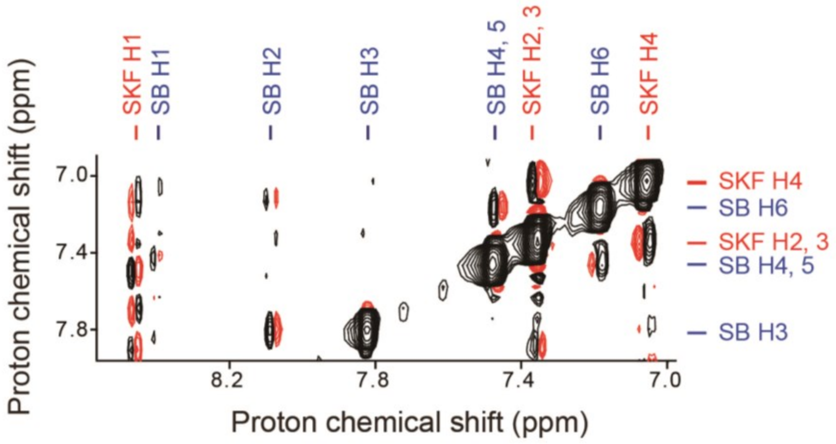

Figure S1. NOESY spectrum of the SKF and SB mixture. The NOESY spectrum was recorded without protein, in the same buffer and under the same experimental conditions as in Figure 2c.

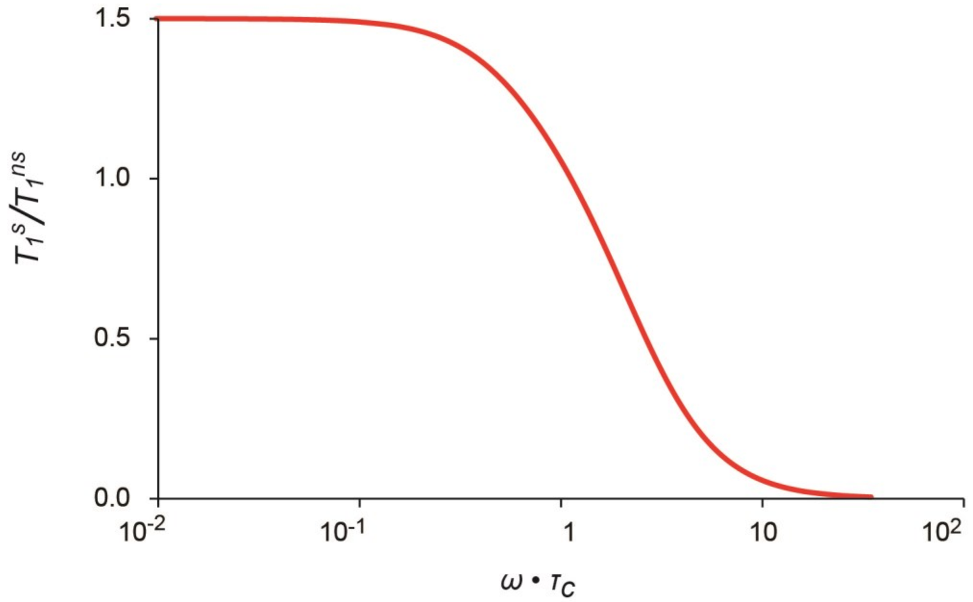

Figure S2. Dependence of the T_1^s/T_1^{ns} values on $\omega{\cdot}\tau_c$. The curve is plotted according to equations (1)–(4).

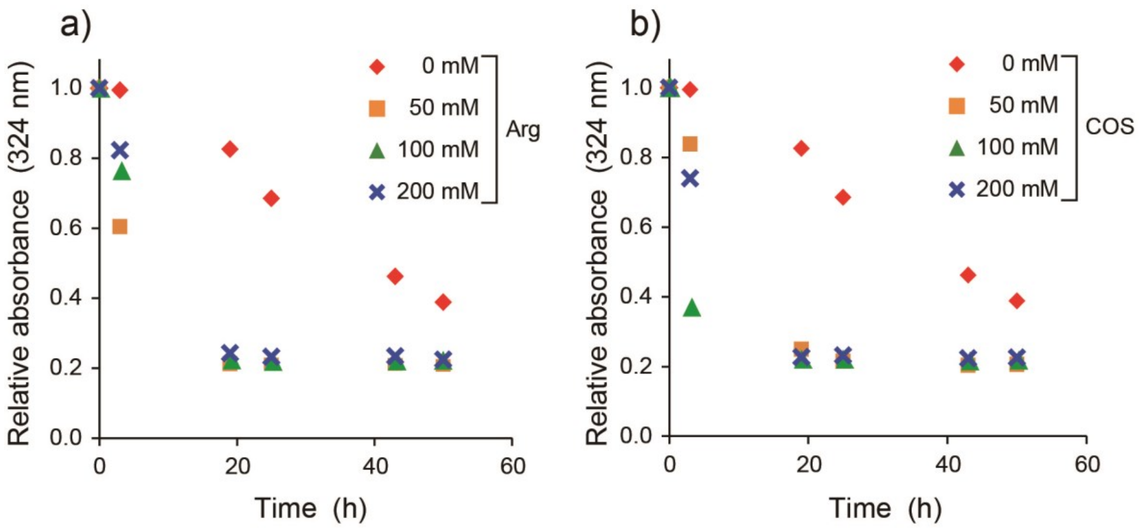

Figure S3. Time-dependent changes in UV absorbance of SKF solution with indicated amount of (a) arginine and (b) COS. The relative absorbance of each sample is plotted against time after sample preparation.

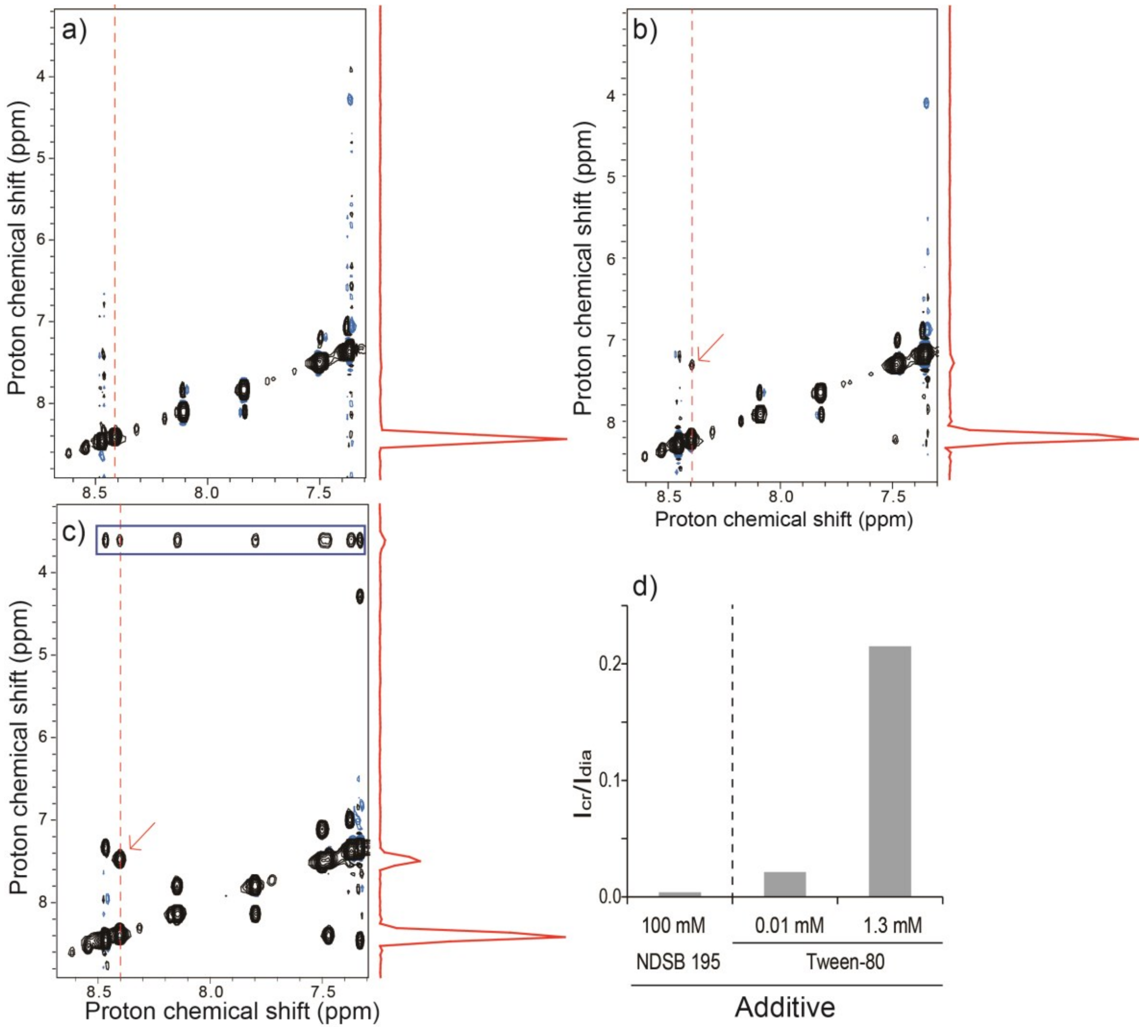

Figure S4. Effect of NDSB or Tween 80 on the NOESY spectra of the SKF SB mixture. The NOESY spectra were recorded for the SKF SB mixture with a) 100 mM NDSB 195, b) 0.01 mM Tween-80, or c) 1.3 mM Tween-80. The experimental conditions, including the concentrations of the compounds, are the same as in Figure 2c without protein. The 1D slices for the SB H1 resonance (red dashed line in the 2D spectra) are shown on the right. The red arrows in b) and c) indicate the intramolecular NOE cross peaks between the SB H1 and the H4,5 protons. The NOE cross peaks between the ligand and Tween-80 protons are enclosed in a box in panel c). d) Intensity ratios of the intramolecular NOE cross peaks between the SB H1 and the H4,5 protons (red arrows in panel b) and c) over the diagonal peak from the SB H1 proton.

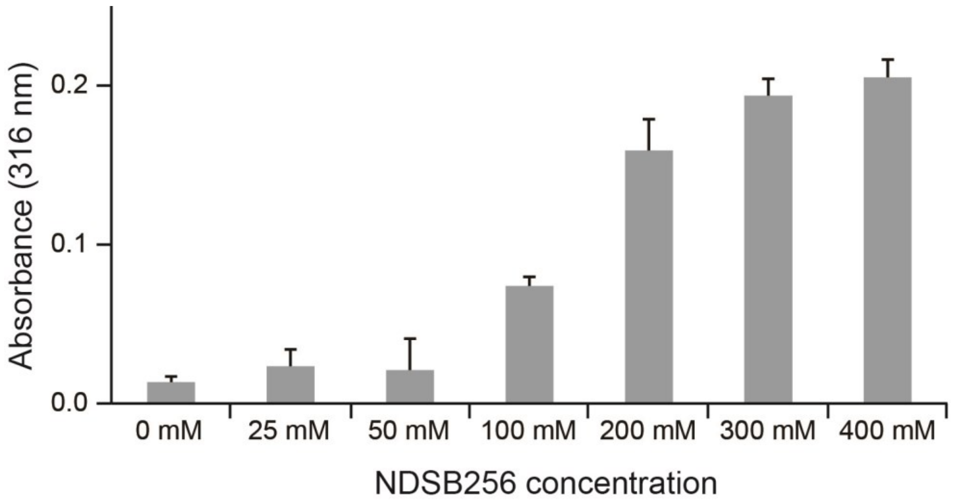

Figure S5. Relief of I4PTH aggregation by NDSB256 co-solubilization. The UV absorbance at 316 nm was monitored for 0.2 mM I4PTH solution with indicated concentration of NDSB256. The absorbance was recorded at 0.5, 1.5, and 2.5 h after sample preparation. The error bars indicate standard deviation of the three measurements, reflecting the stability of the sample.
